# Subtractive inhibition assay for the detection of *Campylobacter jejuni* in chicken samples using surface plasmon resonance

**DOI:** 10.1038/s41598-019-49672-2

**Published:** 2019-09-20

**Authors:** Noor Azlina Masdor, Zeynep Altintas, Mohd. Yunus Shukor, Ibtisam E. Tothill

**Affiliations:** 1 Cranfield University, Cranfield, Bedfordshire, MK43 0AL England United Kingdom; 2 Biotechnology and Nanotechnology Research Center, Malaysian Agricultural Research and Development Institute, MARDI, P. O. Box 12301, 50774 Kuala Lumpur, Malaysia; 3 Technical University of Berlin, Straße des 17. Juni 124, Berlin, 10623 Germany; 4 Department of Biochemistry, Faculty of Biotechnology and Biomolecular Sciences, Universiti Putra Malaysia, 43400 Serdang, Selangor Malaysia

**Keywords:** Surface plasmon resonance, Assay systems

## Abstract

In this work, a subtractive inhibition assay (SIA) based on surface plasmon resonance (SPR) for the rapid detection of *Campylobacter jejuni* was developed. For this, rabbit polyclonal antibody with specificity to *C. jejuni* was first mixed with *C. jejuni* cells and unbound antibody was subsequently separated using a sequential process of centrifugation and then detected using an immobilized goat anti-rabbit IgG polyclonal antibody on the SPR sensor chip. This SIA-SPR method showed excellent sensitivity for *C*. *jejuni* with a limit of detection (LOD) of 131 ± 4 CFU mL^−1^ and a 95% confidence interval from 122 to 140 CFU mL^−1^. The method has also high specificity. The developed method showed low cross-reactivity to bacterial pathogens such as *Salmonella* *enterica* serovar Typhimurium (7.8%), *Listeria monocytogenes* (3.88%) and *Escherichia coli* (1.56%). The SIA-SPR method together with the culturing (plating) method was able to detect *C*. *jejuni* in the real chicken sample at less than 500 CFU mL^−1^, the minimum infectious dose for *C*. *jejuni* while a commercial ELISA kit was unable to detect the bacterium. Since the currently available detection tools rely on culturing methods, which take more than 48 hours to detect the bacterium, the developed method in this work has the potential to be a rapid and sensitive detection method for *C*. *jejuni*.

## Introduction

It is estimated that the yearly medical and productivity losses caused by *Campylobacter* infections are over USD one billion^1^. There are more than 30 species and eleven subspecies in the genus *Campylobacter*. In poultry, the most often found species is *C*. *jejuni*, and it is the leading cause of foodborne illnesses in man. As chicken meat is the top consumed food globally, Campylobacteriosis risk is higher in chicken meat than in other meat and poultry products^2^. Biosensor offers a rapid, sensitive and robust detection methods for pathogen including *C*. *jejuni*^3^. Surface plasmon resonance (SPR) is widely used for the determination of a wide range of analytes, including bacteria *via* direct detection. However, the direct detection of bacteria has some limitations; chiefly it is less sensitive due to the restricted efficient penetration degree of the evanescent field coming up within the circumstances of total internal reflection (TIR), which happens to be roughly 300 nm^4,5^. Bacteria, including *C*. *jejuni* with a size of around 5 µm, exceeds the evanescent field limit. Thus, only a meagre measurable signal can be obtained from a small section of the bacterium^5,6^.

To date, the most sensitive detection of *C*. *jejuni* with SPR platforms showed a limit of detection (LOD) value of 10^2^ CFU mL^−1^ using the receptor binding protein (RBP) of the *Campylobacter* bacteriophage NCTC 12673^7^ followed by using commercial polyclonal antibodies achieving a LOD of 10^3^ CFU mL^−1^ ^6^. A more recent SPR-based method for the detection of *C*. *jejuni* developed by our group yield a LOD value of 4 × 10^4^ CFU mL^−1^ ^5^. Although the first method that relies on *Campylobacter* bacteriophage is sensitive, this bioreceptor is not commercially available, and its production requires a complicated procedure. Hence, the use of antibodies as bioreceptor for the development of *C*. *jejuni* detection is still a major choice for food samples analysis. However, due to the limitations arising from SPR penetration depth in case of detecting large pathogens, the results generally lack sensitivity. The penetration depth generally does not allow performing sandwich assays with desirable LOD as it increases the height of the sensor surface further.

**Figure 1:**
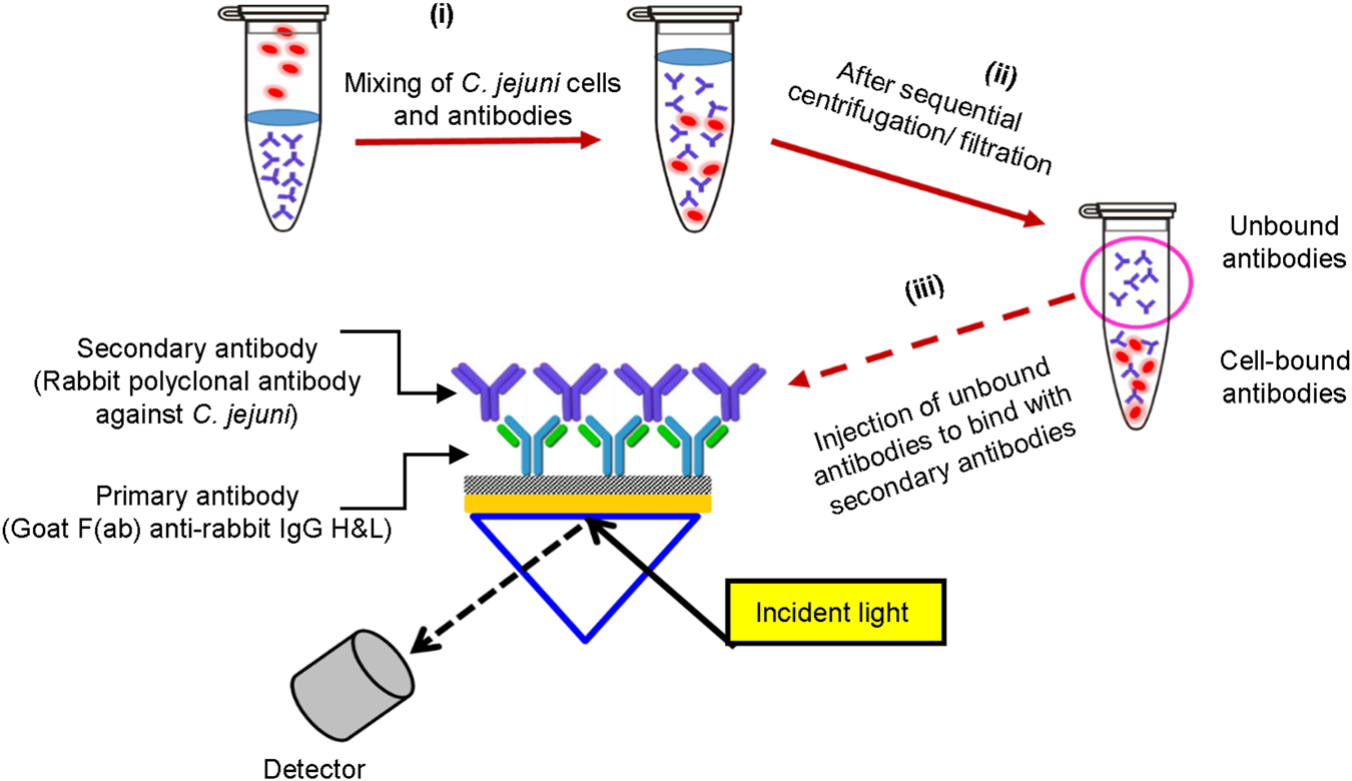
Schematic of the subtractive inhibition assay format.

An emerging technique to overcome this problem in SPR-based detection of *C*. *jejuni* is the subtractive inhibition assay. This method (Fig. 1) progress with an initial mixing of antibody and bacterial cells, followed by the separation of the unbound from the cell-bound antibodies via sequential centrifugation and finally, the remaining unbound antibody is quantified through the interaction with a previously immobilized anti-antibody on the SPR sensor chip surface^8^. As the size of the antibody is within the penetration depth of the evanescent field, this boosts the level of sensitivity of the SPR for the detection of bacteria^9–12^.

In the present work, a subtractive inhibition assay to develop a sensitive SPR-based immunosensor for the detection of *C*. *jejuni* using a rabbit polyclonal antibody with specificity to *C*. *jejuni* is reported for the first time. Highly sensitive and specific quantification of this bacterium is successfully achieved using this approach to the best of our knowledge.

## Results

### Primary capture antibody concentration optimization

**Figure 2:**
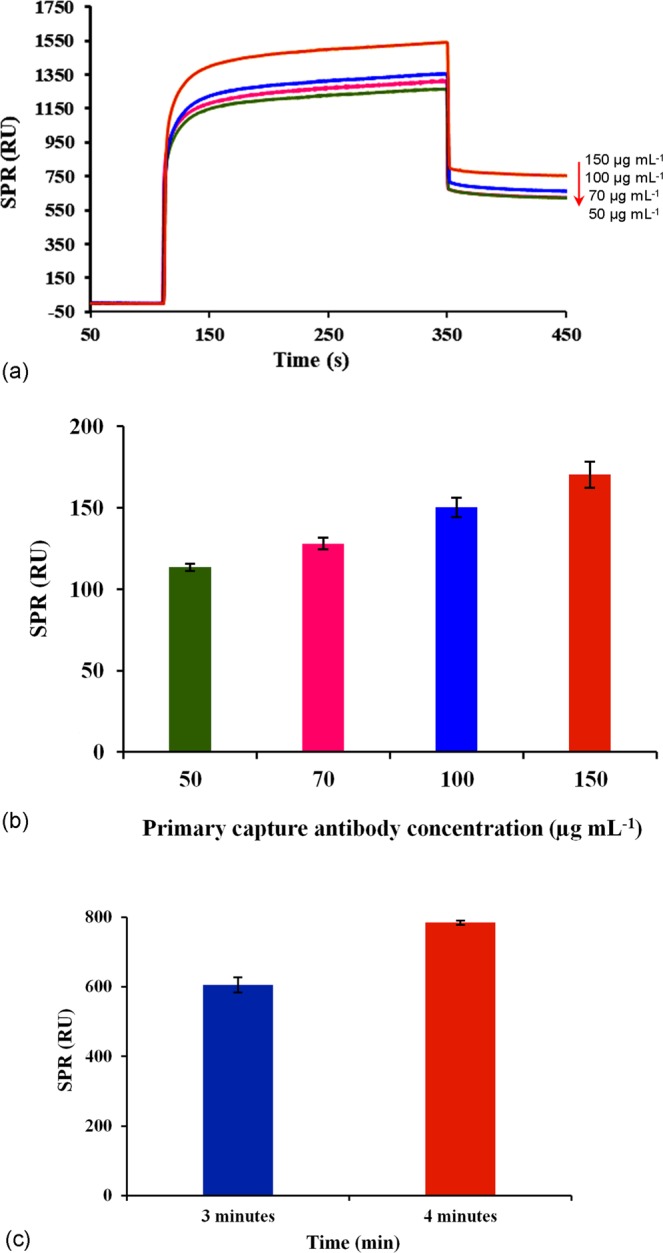
Sensorgram for optimization of antibody immobilization using different concentrations of primary capture antibody (goat F(ab) anti-rabbit IgG H&L antibody). (**a**) Sensor responses obtained due to the binding of 100 µg mL^−1^ of the secondary a rabbit polyclonal antibody with specificity to *C*. *jejuni*. (**b**) Comparison of the binding response obtained from 3 and 4 minutes of injection period of the primary capture antibody during the immobilization process. (**c**) Error bars represent the mean ± standard deviation of triplicates.

The optimal concentrations of primary and secondary antibodies are vital to achieve a maximum binding response. In order to optimize this step, various concentrations of the primary antibody (goat F(ab) anti-rabbit IgG H&L antibody) at 50, 70, 100 and 150 µg mL^−1^ were first immobilized on the SPR sensor chip followed by the injection of 100 µg mL^−1^ of the secondary a rabbit polyclonal antibody with specificity to *C*. *jejuni*. The sensorgram in Fig. 2a shows that the immobilization response changed with the increasing concentration of the primary antibody, and Fig. 2b displays the binding response of 100 µg mL^−1^ secondary antibody at each primary antibody concentration used. From these results, the primary antibody concentration of 150 µg mL^−1^ gave the highest signal responses for both antibody immobilization and secondary antibody capture. ANOVA analysis of the results show that there was a significant difference (p < 0.05) in the immobilization response obtained from the primary antibody at 150 µg mL^−1^ compared to other antibody concentrations. Hence, 150 µg mL^−1^ was chosen as the optimum concentration for immobilization of the primary antibody. Higher concentrations of primary antibody were not tested as this would not be economical. In addition, the binding response obtained at 150 µg mL^−1^ was considered satisfactory. Further investigation of the injection period shows that a 4 min (100 µL) injection period gave a significantly higher response (p < 0.05) than a 3-min injection (75 µL). Hence, a 4 min (100 µL) injection was chosen for further optimization studies (Fig. 2c).

### Optimization of the free unbound secondary antibody separation technique

**Figure 3:**
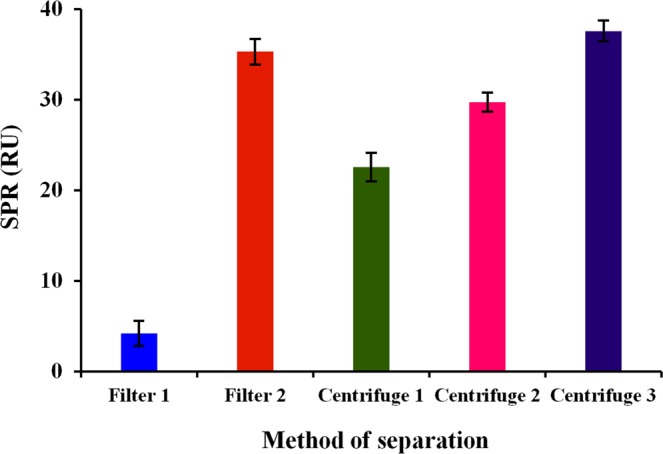
Comparison of unbound antibody separation process using filtration and centrifugation techniques. The filtration technique utilizes syringe filters with the MWT cutoffs of 0.1 and 0.2 µm. The centrifugation technique consists of a direct centrifugation at 3200 × g for 1 min, a sequential centrifugation (200, 400, 800, 1200, 1600 and 3200 × g) with each step lasting for 1 min and a sequential centrifugation (200, 400, 800, 1200, 1600 and 3200 × g) with each step lasting for 2 min. Error bars represent the mean ± standard deviation of triplicates.

In a subtractive inhibition assay, either filtration or a centrifugation method with a single or a sequential step has been employed to separate the free unbound secondary antibodies. Thus, all of the techniques were explored. The results in Fig. 3 show that the best technique giving the highest inhibition value (37.6 RU) was a sequential centrifugation step lasting for 2 min. The second-best method was with the filtration step by employing a filter possessing a MWT cut-off of 0.2 µm, and this led to an SPR signal of 35.1 RU. The lowest result was obtained with a filtration using a 0.1 µm MWT cut-off filter that provided an inhibition value of 4.2 RU.

### Optimization of secondary antibody binding to *C*. *jejuni* cells

**Figure 4:**
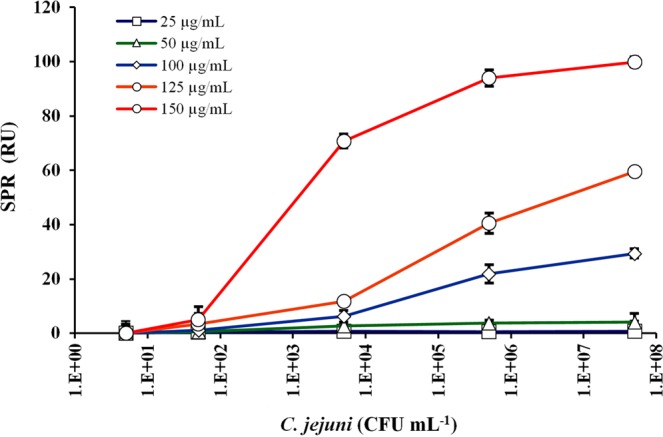
The optimization of secondary antibody concentration binding to *C*. *jejuni* cells at five different concentrations at 50,100, 200, 250 and 300 µg mL^−1^ that were mixed with a series of *C*. *jejuni* cells concentrations at 0, 10, 10^2^, 10^4^, 10^6^ and 10^8^ CFU mL^−1^. Error bars represent the mean ± standard deviation of triplicates.

The results in Fig. 4 show that 150 µg mL^−1^ was the best secondary antibody concentration exhibiting the highest binding response of about 100 RU at 5 × 10^7^ CFU mL^−1^ concentration of *C*. *jejuni*. The second-best concentration for secondary antibody was 125 µg mL^−1^ with an SPR signal of 59.5 RU at 5 × 10^7^ CFU mL^−1^, which was about 40% lower than the response observed using 150 µg mL^−1^. Remarkably, this difference is even more pronounced at lower *C*. *jejuni* cell concentrations. For example, at 5 × 10^3^ CFU mL^−1^, the binding responses obtained with 150 µg mL^−1^ and 125 µg mL^−1^ secondary antibody were 70.74 and 11.8 RU, respectively. This is about an 83% reduction of the signal.

### Direct subtractive inhibitive immunoassay for detection of *C*. *jejuni*

**Figure 5:**
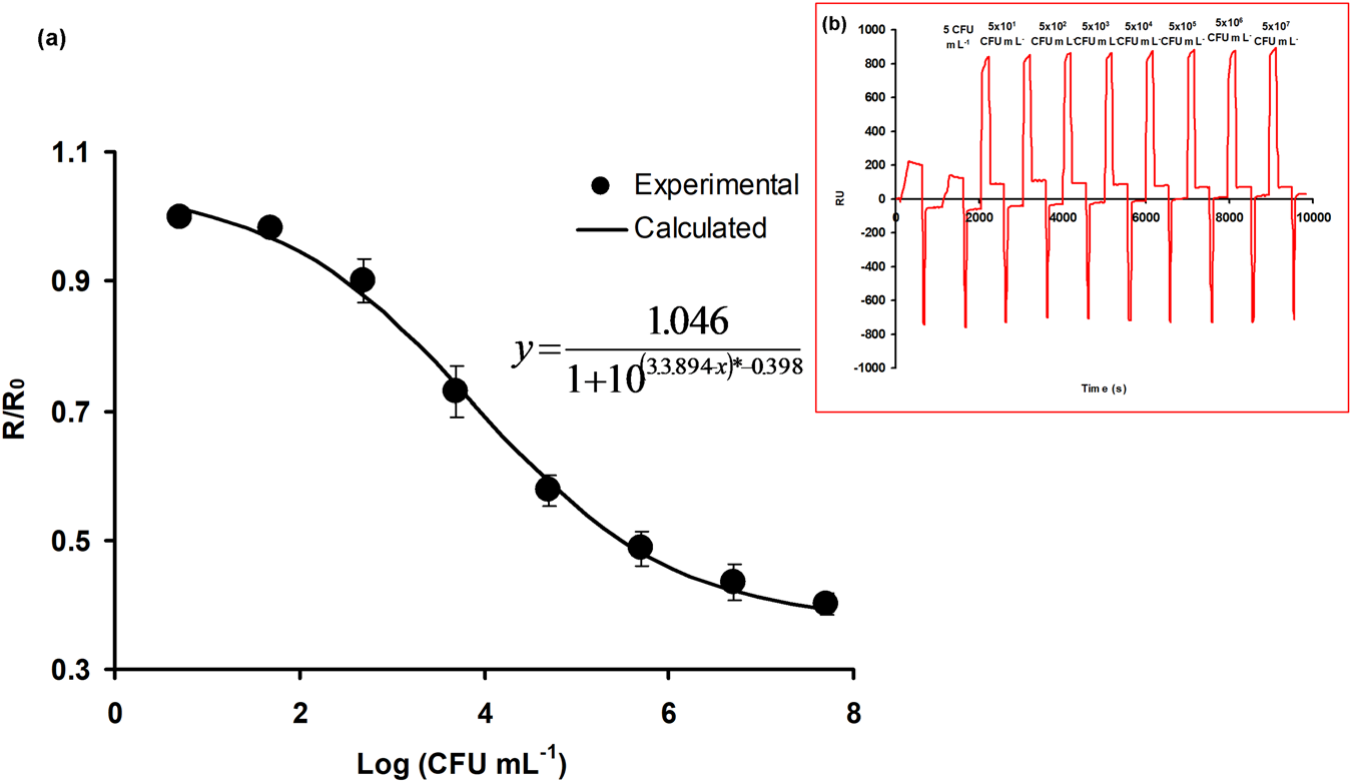
Plot of normalised response (R/R_0_) versus the concentration of *C*. *jejuni* ranging from 5 to 5 × 10^7^ CFU mL^−1^ (**a**) and concentration-dependent sensorgram displaying the binding response of various concentrations of *C*. *jejuni* (**b**) obtained through subtractive inhibition assay. Error bars represent the mean ± standard deviation of triplicates.

The sensorgrams in Fig. 5a shows the direct binding of *C*. *jejuni* to the immobilized capture secondary antibody over the entire calibration range from 5 to 5 × 10^6^ CFU mL^−1^ and control (PBS). The data was transformed and normalized into the ratio *R*/*R*_0_, which is each sample average response divided by the value of the average control^9^. As expected, an inverse relationship was observed between the binding response and concentrations of *C*. *jejuni* cells, which is a verification of the subtractive inhibition assay.

An inverse sigmoidal dose response was observed in Fig. 5b, and the most appropriate model to be utilized under this circumstance is the same four parameter logistics model (Karpinski model) with modifications to the standard formula. A gradual increase in the response unit was observed as the concentrations of *C*. *jejuni* was increased. The calculated LOD value was 131 ± 4 CFU mL^−1^ and a 95% confidence interval from 122 to 140 CFU mL^−1^.

### Cross-reactivity against other bacteria

**Figure 6:**
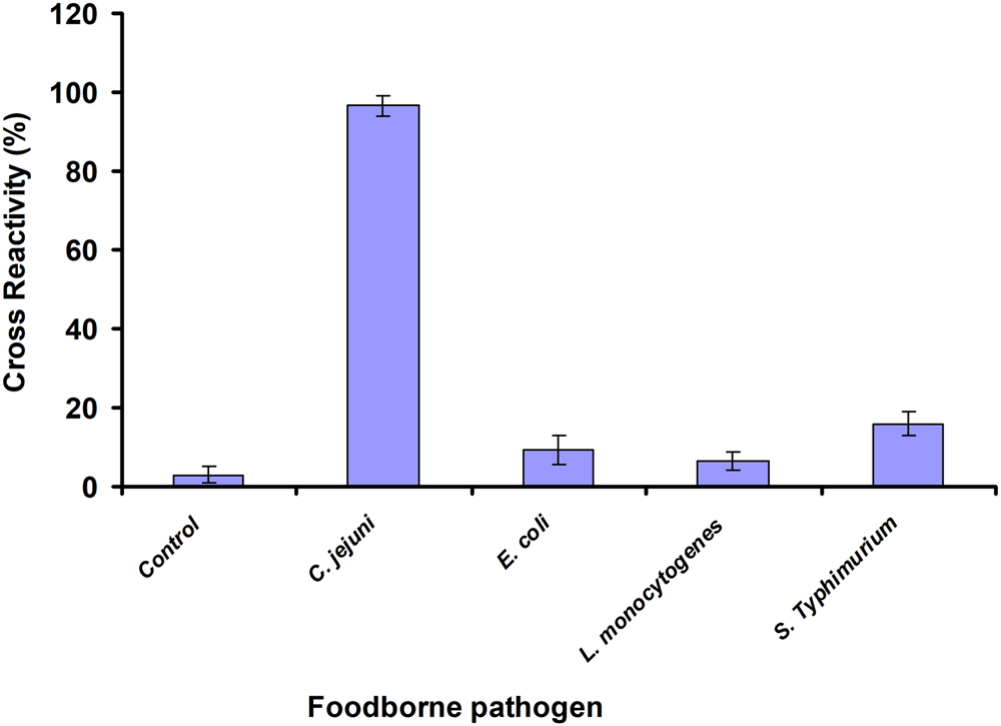
Specificity of developed *C*. *jejuni* assay compared with other foodborne bacteria, *E*. *coli*, *L*. *monocytogenes*, *Salmonella* *enterica* serovar Typhimurium and control (PBS) using the subtractive inhibition assay. (**a**) Error bars represent the mean ± standard deviation of triplicates.

A specificity study for the developed subtractive inhibition assay format was carried out using three common foodborne pathogens, which were *L*. *monocytogenes*, *E*. *coli*, and *Salmonella* *enterica* serovar Typhimurium. *E*. *coli* exhibited the lowest cross-reactivity at 1.55% while *Salmonella* *enterica* serovar Typhimurium exhibited the highest cross-reactivity at 7.8% (Fig. 6).

### Detection of *C*. *jejuni* in chicken samples

Out of the two samples (A1 and A2), only sample A1 was positive for *C*. *jejuni*. The presence of *C*. *jejuni* in sample A1 was also detected by the SPR while the ELISA kit failed to detect the presence of the bacterium in the A1 sample (Table 1).

**Table 1:** Comparative study for *C*. *jejuni* determination in chicken samples using the plating method, SPR and using a commercial ELISA kit.

Sample code	Culturing method (plate) CFU mL^−1^	SPR		ELISA kit	
		Signal response (*R*/*R*_0_)	Number of cells (based on correlation of the standard curve)-CFU mL^−1^	Abs at 450 nm	Remarks *Any value >0.1 considered as positive
A1	140 ± 20	0.868 ± 0.040	3.26 × 10^2^ ± 1.03 × 10^2^	0.046 ± 0.012	Negative
A2	0	0.980 ± 0.015	Negative	0.047 ± 0.012	Negative

Data represent the mean ± standard deviation of triplicates.

## Discussion

One of the drawbacks of bacterial detection using SPR is the problem of the penetration depth of the evanescent wave, which is limited. A real solution to this issue is using the subtractive inhibition assay, which has shown the applicability in improving LOD of bacterial detection over several orders of magnitude^13^. In this study, we explored the subtractive inhibition assay as a method to improve the LOD of *C*. *jejuni* on an SPR platform. One of the most critical key aspects of SIA is the optimization of the separation of bound from unbound antibodies. This study shows that the centrifugation method via a sequential approach is the most optimum. Other SPR works showed various methods for the separation of bound from unbound primary antibodies including separation through a 0.22 μm MWT cut-off filter^10^. However, the filtration step loses substantial antibody amount as a portion of the antibody is trapped to the body of the syringe filter, making this technique costly. The rest of the subtractive inhibition assay methods utilize centrifugation technique either in single or sequential forms. For instance, Leonard *et al*.^11^ separated bound and unbound primary antibodies against *L*. *monocytogenes* cells by a sequential centrifugation step of 50, 200, 450, 800, 1200, 1800 and 3200 × g at 1 min interval. They reported that a single centrifugation step of 1800 × g gave poor results. This stepwise centrifuging process is also utilized by Wang *et al*.^9^, Wang *et al*.^12^ and Kong *et al*.^13^ in the detection of *B*. *anthracis* A16, *E*. *coli* O157:H71 and *B*. *cereus*, respectively. On the other hand, a single centrifugation step is utilized in the detection of *Porphyromonas gingivalis*^14^.

The optimization results for the amount of antibody show a substantial difference in terms of response at lower bacteria concentrations indicated that this part is one of the most important optimization steps in the subtractive inhibition assay. However, nearly all of the published research works on SPR that use the subtractive inhibition assay with antibody as a bioreceptor^9,11,12^ did not report optimization results for the secondary antibody. As this step requires the use of a high amount of antibody, this is probably the reason why this step is overlooked or not given adequate attention. If this step had been optimised, a vast improvement of LOD could probably be seen. In the current work, higher concentrations than 150 µg mL^−1^ were not tested due to economic reasons, as well as the signal obtained of about 100 RU is considered satisfactory. The maximum binding response observed in this work is comparable to several SPR-based pathogen assays. As examples, Wei *et al*.^6^ reported a binding response of 85 RU for 1 × 10^7^ CFU mL^−1^ of *C*. *jejuni*; Waswa *et al*.^15^ reported 100 RU binding response for 1 × 10^8^ CFU mL^−1^ *S*. *enteritidis* and 1 × 10^7^ CFU mL^−1^ *E*. *coli* in their work. Another research demonstrated a binding response of 100 RU for 1 × 10^6^ CFU mL^−1^ *Salmonella* *enterica* serovar Typhimurium^16^.

A few SPR works reported the optimal secondary antibody concentrations for subtractive inhibition assay. For instance, Wang *et al*.^12^ utilize 10 µg mL^−1^ of mouse monoclonal antibody against *B*. *anthracis* A16 spores as the secondary antibody while in the detection of *E*. *coli* O157:H71 Wang *et al*.^9^ utilize 25 µg mL^−1^ of the secondary antibody. Although the antibody concentrations used in their work are much lower than in the current study, the LOD values reported in their work are less sensitive and much higher than our work. In another subtractive inhibition assay work, Leonard *et al*.^11^ utilize 300 µL of a 1/250 dilution of protein-G purified anti-*L*. *monocytogenes* antibody without reporting the exact amount of secondary antibody utilized.

The subtractive inhibition assay gave a very good LOD value of 131 CFU mL^−1^, suggesting that the developed assay is very sensitive. In comparison, a direct immunoassay for detection of *C*. *jejuni* using the same polyclonal antibody in this work as a bioreceptor on the same SPR instrument gave a LOD value of 2 × 10^5^ CFU mL^−1^ ^5^, which is anticipated due to the limited penetration depth issue. To date, the subtractive inhibition assay developed in this work is one of the most sensitive SPR-based assays for *C*. *jejuni* and is comparable to the results of Singh *et al*.^7^ using a *C*. *jejuni* bacteriophage in a direct format for the detection of the bacterium with a LOD of 1 × 10^2^ CFU mL^−1^. Another SPR-based assay for this pathogen showed LOD values of 1 × 10^3^ CFU mL^−1^ ^6^ and 1 × 10^5^ CFU mL^−1^ ^17^. As far as the LOD value for subtractive inhibition assay in an SPR format is a concern, the LOD value reported is the most sensitive one. The current most sensitive subtractive inhibition assay in an SPR format is for the detection of *B*. *cereus* with a LOD of 1 × 10^2^ CFU mL^1^ ^13^. Other subtractive inhibition assay-based method in SPR instrument showed an average LOD improvement of one order of magnitude^9,11,12^ or no improvement in LOD^14^, while Kong *et al*.^13^ and this work showed an improvement of three orders of magnitude.

The results for the cross-reactivity study indicate the developed assay has low cross-reactivity to other bacterial pathogens, which is almost the same to the results using whole-cell detection on SPR previously developed using the same polyclonal antibody^5^. A varying range of cross-reactivity is also reported in numerous immunosensor methods for detecting *C*. *jejuni*. For example, an indirect ELISA assay to determine *C*. *jejuni* based on polyclonal antibody shows low cross-reaction of less than 10% to other bacteria such as *Salmonella* *enterica* serovar Typhimurium, *E*. *coli*, *S*. *enteritidis*, *Enterococcus faecalis*, *Yersinia pestis*, *Bacillus cereus* and *Bacillus subtilis*^18^, indicating that a polyclonal antibody preparation can be very specific. Substantial cross-reactivity was observed in the work of Wei *et al*.^6^ where a high cross-reactivity of about 44% at 10^4^ CFU mL^−1^ and 70% at 10^6^ CFU mL^−1^ was observed when the developed immunosensor was challenged by *Salmonella* *enterica* serovar Typhimurium. Wei *et al*.^6^ also challenged their developed system with other species of the *Campylobacter* genera such as *C*. *coli* and *C*. *lari* and found minimal cross reactivity.

In another SPR-based detection method for *C*. *jejuni*, cross-reactivity studies with three other bacterial pathogens: *E*. *coli* O157: H7, *Salmonella* *enterica* serovar Typhimurium, and *Listeria monocytogenes* showed a cross-reactivity value of about 25%^17^. Several other works on *C*. *jejuni* detection using QCM with lectin as the bioreceptor did not investigate the cross-reactivity^19,20^. Low to negligible cross-reactivities are reported for the detection of bacteria using SPR with the non-antibody type of bioreceptors. For instance, negligible cross reactivity is reported for the detection of *Staphylococcus* aureus in the presence of *Salmonella* *enterica* serovar Typhimurium using lytic phage as the bioreceptor^21^, while Yazgan *et al*.^22^ reported a low level of cross reactivity in the detection of *E*. *coli* to the bacteria *Citrobacter freundii* and *Staphylococcus epidermidis* at 2.6% and 8.6%, respectively, using mannose-containing oligosaccharides as the bioreceptor. There seems to be no consensus on what percentage of cross-reactivity is considered too high or too low. The consensus cut-off threshold value would be dependent upon acceptable false negatives or positives level decided for a given diagnostic application.

The application of a developed immunosensor on real-world samples is one of the most important activities for the validation of the sensor. Validation with real samples holds important consequences and benefits. This includes a compliance process for the developed immunosensor to be accepted as a standard method such as ISO, and this is required by enforcement agencies in many countries. Field trial works using real-world samples would also allow risk assessments activity to be developed, and remedial actions to be taken based on the immunosensor. Validation of any developed immunosensor require a benchmarking with regulatory bodies-accepted method, and in the case of *C*. *jejuni* detection in chicken samples, it is the culturing method which is endorsed by regulatory bodies and standard methods such as the International Organisation for Standardisation (ISO), UK-based Chilled Food Association (CFA)/British Retail Consortium (BRC), and many others^23^. However, recently published works have shown that the culturing method is not enough in distinguishing *C*. *jejuni* from other *Campylobacter* genera such as *C*. *coli*, *C*. *lari* and *C*. *upsaliensis* and genotyping methods which include a comparative analysis of the 16S rRNA gene sequences should be added to confirm the identity^24–26^.

The failure of the ELISA method to detect any *C*. *jejuni* cells in both samples is anticipated as the kit is unable to detect *C*. *jejuni* cells at less than 10^6^ CFU mL^−1^ ^5,27^. The plating or the culturing method was successful in detecting *C*. *jejuni* in a real sample. However, this does not mean that there is no other bacterium from the genera *Campylobacter* present in the samples^28^ as their detection is beyond the scope of this work. Both the culturing method and conditions as used in this experiment are utilised in the detection of *C*. *jejuni* in chicken samples via SPR by Wei *et al*.^6^ and by fluorescent immunoassay by Wang *et al*.^29^. On the other hand, the culturing method has not been utilized as a benchmark for the detection of *C*. *jejuni* in some works^17,30,31^. The results obtained in this work demonstrate the applicability of the SPR-SIA method to rapidly detect *C*. *jejuni* at the comparable level of the gold standard plating method. In addition, the results also show the weakness of the current ELISA approach as a sole method in detecting *C*. *jejuni* in food samples.

The infectious dose for *C*. *jejuni* is around 500 CFU mL^−1^, and the SIA-SPR method can detect the presence of live bacterium at the infectious dose making the method suitable as a rapid and sensitive method to detect *C*. *jejuni* as a rapid early warning method, and with the time-consuming plating method as a further confirmatory method. This approach can save lives and reduce disease as a result of consuming *C*. *jejuni*-contaminated foods. Further works are necessary to validate the use of the SPR method as an early warning method by increasing the number of real food samples and adding another confirmatory method such as molecular (PCR) to strengthen the results.

## Methods

### Materials

A rabbit polyclonal antibody with specificity to *C*. *jejuni* was sourced from MARDI (the Malaysian Agricultural Research and Development Institute), Malaysia. Goat F(ab) anti-rabbit IgG H&L (ab6824) were purchased from Abcam Ltd., UK. N-hydroxysuccinimide (NHS) were sourced from Thermo Scientific, UK. 11-mercaptoundecanoic acid (11-MUDA), 1-ethyl-3-(3-dimethylaminopropyl)-carbodiimide (EDC), ethanolamine hydrochloride, ethanol, hydrochloride acid (HCl), sodium acetate, PBS (phosphate buffered saline tablet, pH 7.4), hydrogen peroxide (H_2_O_2_), sulfuric acid (H_2_SO_4_), bovine serum albumin (BSA) were purchased from Sigma-Aldrich, UK. All methods were carried out in accordance with relevant guidelines and regulations. All experimental protocols were approved by the Malaysian Agricultural Research and Development Institute and Cranfield University, and carried out in accordance with the relevant guidelines and regulations.

### Preparation of *C*. *jejuni* cells and other bacterial strains

The maintenance, growth and preparation of heat-killed *C*. *jejuni* (*C*. *jejuni* subsp. *jejuni* ATCCs 33291) were carried out as reported elsewhere^32^. Xylose lysine deoxycholate agar (XLD), Oxford medium and MacConkey sorbitol agar (Acumedia Manufacturers Inc., Baltimore, MD, USA) were utilized to enumerate *Salmonella* *enterica* serovar Typhimurium, *L*. *monocytogenes*, and *E*. *coli* O157: H7, respectively. *Salmonella* *enterica* serovar Typhimurium, *Listeria monocytogenes* and *E*. *coli* O157: H7 were obtained from the culture collection of MARDI. *Campylobacter jejuni* subsp. *jejuni* ATCC® 33291 was sourced from Fischer Scientific, UK.

### Instrumentation

A fully automated SPR-4 biosensor with its amine coated chips from Sierra Sensors, GmbH, Germany was employed in the immunosensor development for *C*. *jejuni* detection. The flow rate of the running buffer and the operating temperature during the assays were 25 µL min^−1^ and 25 °C, respectively. Data from the SPR-4 were further analysed with Microsoft Excel and the R2 software from Sierra Sensors (Hamburg, Germany).

### Subtractive inhibition assay setup

Two types of antibodies; a ‘primary antibody’ which was a goat F(ab) anti-rabbit IgG H&L and a rabbit polyclonal antibody with specificity to *C*. *jejuni* as the ‘secondary antibody’ were utilized in the development of the subtractive inhibition assay. The interaction between the primary and the injected secondary antibodies was quantified in a direct assay via SPR (Fig. 1).

### Optimization of the primary antibody concentration

An antibody of goat F(ab) anti-rabbit IgG H&L was dissolved in sodium acetate (100 mM, pH 4.5) at varying concentrations. Various concentrations of the capturing antibody (50, 70, 100 and 150 µg mL^−1^) were injected for 4 min across the sensor surface at a flow rate of 25 µL min^−1^. A secondary antibody which was rabbit polyclonal antibody with specificity to *C*. *jejuni* (100 µg mL^−1^) was then injected over the immobilized capture antibody on the sensor surface for 4 min (75 µL).

### Surface activation and antibody immobilization

The running buffer for this experiment is a filtered and degassed PBS (0.01 M, pH 7.4) and was utilized at a constant flow rate of 25 μL min^−1^ until a stable baseline was achieved. The antibody immobilization on the gold sensor surface commenced with the activation of the MUDA coated surface using an EDC-NHS solution, which was injected at a flow rate of 25 μL min^−1^ for a total duration of 3 min^33^. Next, an optimized concentration of primary capture antibody of 150 µg mL^−1^ was injected for 4 min (100 µL). The sensor spot was then blocked by utilizing a 50 μg mL^−1^ injection (4 min, 75 µL) of BSA dissolved in PBS. Unreacted NHS esters were capped by utilizing injection of 1 M of ethanolamine (pH 8.5) for 4 min (75 µL). Recording of the change in response (RU) after protein injection was recorded for 2 min after the completion of the injection^34^.

### Optimization of the free unbound secondary antibody separation technique

A 300 µL of 200 µg mL^−1^ secondary antibody (a rabbit polyclonal antibody with specificity to *C*. *jejuni*) prepared in PBS was mixed with 300 µL of 1 × 10^8^ CFU mL^−1^ of *C*. *jejuni* cells on a roller (Stuart SRT6, Keison International Ltd., UK) for 1 hour at room temperature. The remaining unbound secondary antibodies in the mixture were then separated from the cells bound antibodies using two different techniques: centrifugation and filtration. In a sequential centrifugation technique, increasing centrifugation forces of 200, 400, 800, 1200, 1600 and 3200 × g was applied in a centrifuge (Heraeus^TM^ Megafuge 8R, Thermo Scientific Inc, US) with each step lasting for 1- or 2-min intervals. In another centrifugation technique, a single centrifugation force of 3200 × g was employed for 1 min. In the filtration technique, two sizes of Minisart cellulose citrate syringe (0.1 and 0.2 µm) were employed (Sartorius, Germany). Appropriate controls were carried out for each of the separation technique employed in this work. The remaining free unbound secondary antibodies were obtained by withdrawing 500 µL supernatants fluids from each of the separation techniques. The remaining free unbound antibodies from each set of experiments were quantified by injecting 75 µL (4 min) of the supernatant fluid over the capture antibody (goat F(ab) anti-rabbit IgG H&L) which has been immobilized previously on the SPR-4 sensor surface. Post-injection surface regeneration of each cycle of the unbound antibodies binding was carried out using 25 µL of 100 mM HCl for 1 min. The control studies were included in each experimental set, and the binding response was subtracted from the control values to get the total inhibition values.

### Optimization of secondary antibody concentration

Secondary antibody at various concentrations (50 to 300 µg mL^−1^) was prepared in 300 µL of PBS and mixed with 300 µL of *C*. *jejuni* cells (0, 1 × 10^2^, 1 × 10^4^, 1 × 10^6^ and 1 × 10^8^ CFU mL^−1^) in a rotating movement on a roller for 1 hour at room temperature. The control experiment was carried out by mixing 300 µL of secondary antibody at various concentrations (50 to 300 µg mL^−1^) and by replacing the bacterial cells with 300 µL of PBS. Free remaining unbound secondary antibodies in the mixture were separated from the cell-bound antibodies using the sequential centrifugation technique above with each step lasting for 2 min. The remaining free unbound secondary antibodies in the supernatant (500 µL) from each set of experiments were quantified by injecting 75 µL (4 min) of the supernatant fluid over the primary capture antibody which has been immobilized previously on the SPR-4 sensor surface. The surface was regenerated after each cycle of secondary antibody binding for subsequent testing. Control studies were included in each experimental set, and the binding response was subtracted from the control values to get the total inhibition values.

### Direct subtractive inhibition assay for the detection of *C*. *jejuni*

The direct detection strategy for *C*. *jejuni* using subtractive inhibition assay was conducted by injection of the remaining free unbound secondary antibodies obtained from a 1-hour incubation of *C*. *jejuni* with 150 µg mL^−1^ secondary antibody (polyclonal antibody against *C*. *jejuni*). In this experiment, a 300 µL of 300 µg mL^−1^ secondary antibody prepared in 1:1 chicken sample matrix was mixed with 300 µL of *C*. *jejuni* cells concentration ranging from 1 × 10^1^ to 1 × 10^7^ CFU mL^−1^. A control experiment was carried out by mixing 300 µL of 300 µg mL^−1^ of antibody and replacing the bacterial cells with 300 µL of PBS. Each mixture was incubated in a roller for 1 hour at room temperature. The free remaining of unbound secondary antibodies in the mixture was separated from the cells bound antibodies using the sequential centrifugation technique above with each step lasting for 2 min. The remaining free unbound antibodies from each set of experiments were quantified by injecting 75 µL (4 min) of the supernatant fluid over the primary capture antibody which was previously immobilized on the SPR-4 sensor surface. The data obtained were transformed into the *R*/*R*_0_ ratio, which is the mean response for each sample divided by the mean control value^9^.

### Specificity of the assay to *C*. *jejuni*

The Gram-negative bacteria; *E*. *coli* O157: H7 and *Salmonella* *enterica* serovar Typhimurium, and the Gram-positive *Listeria monocytogenes* were utilized to study the specificity of the developed subtractive inhibition assay. A 300 µL of 200 µg mL^−1^ secondary antibody (a rabbit polyclonal antibody with specificity to *C*. *jejuni*) prepared in PBS was mixed with 300 µL of different bacteria placed in different tubes at the concentration of 1 × 10^6^ CFU mL^−1^. The control for this experiment was carried out by mixing 300 µL of 200 µg mL^−1^ of antibody and replacing the bacterial cells with 300 µL of PBS. All of the mixtures were then incubated and centrifuged with the same optimized method, as mentioned previously. The remaining free unbound secondary antibodies from each set of experiments were quantified by injecting 75 µL (4 min) of the supernatant fluid over the capture antibody, which was initially immobilized on the sensor surface. The percentage of cross-reactivity in the direct subtractive inhibition assay was calculated as the following where BR represents the binding response:

$$ \% Cross-reactivity=\frac{BR\,of\,control-BR\,of\,bacterium}{BR\,of\,control-BR\,of\,C.\,jejuni}\times 100$$

### Determination of limit of detection for subtractive inhibition assay

In the subtractive inhibition assay, the calibration curves were fitted with a non-linear regression using a four-parameter dose-response equation^35^ as follows:

$$y=\frac{d+(a-d)}{1+{10}^{(c-x)b}}$$

where ***y*** represents the response signal (RU), ***x*** represents the bacterial cell concentration (log CFU mL^−1^), ***a*** and ***d*** represent the maximum and minimum signal response of the calibration curve respectively, ***b*** is the Hill coefficient which represents the slope-like parameter and ***c*** represents the bacterial cells concentration (log CFU mL^−1^) producing a 50% signal response (EC_50_) value. A statistically robust method for calculating the analytical LOD of a typical sigmoidal relationship was utilized^36^. Another requirement is the standard deviations must be homoscedastic. Test for homoscedasticity was carried out using the Bartlett and the Levene’s tests^37^ and was found to satisfy the requirement of homoscedasticity, a requirement for the LOD determination based on the pooled standard deviation (PSD) to work. The limit of detection (LOD) was calculated as the mean value of absorbance at a blank concentration of bacteria at three PSD. The four-parameter logistics model was utilized to calculate the LOD and regression analysis using the non-linear regression analysis software PRISM (v 5.1) from www.graphpad.com.

### Preparation of chicken sample

Two chicken samples were bought from two local retail outlets in Milton Keynes, UK. Boneless chicken sample (25 g) was added to a sterile Baxfilter™ bag from Interscience (France) containing 225 mL of Bolton broth. The bag was then shaken vigorously by hand for 1 min. From each of the Baxfilter™ bag, 100 mL of the filtered chicken rinse samples were transferred to sterile dilution bottles for further analysis. The rinse was analysed for the presence of *C*. *jejuni* by plating 100 µL aliquots of the rinse in triplicates on Campy Cefex agar. Plates were incubated in an anaerojar under microaerophilic conditions at 42 °C for 48 hrs. The presence of *C*. *jejuni* was observed through the colony and morphological properties obtained on the agar.

### Detection of *C*. *jejuni* in chicken samples using SIA

Preliminary results show that a 1:1 dilution of the chicken rinse and the use of 300 mM NaCl (final concentration) were the best conditions for minimizing chicken sample matrix non-specific binding effect. The chicken rinse sample was diluted in a 1:1 ratio by mixing the rinse with NaCl to a final concentration of 300 mM in a final volume of 300 µL. This is followed by mixing the sample with 300 µL of 300 µg mL^−1^ secondary antibody prepared in PBS (10 mM, pH 7.4). The final volume was 600 µL.

The mixture was then gently rotated, rotating on a roller (Stuart SRT6, Keison International Ltd., UK) for 1 hr at room temperature. This allowed for a maximum mixing of the antibody and chicken sample containing NaCl and *C*. *jejuni* cells. The free remaining unbound antibodies were then separated from cells bound antibodies by utilizing a series of sequential centrifugation forces of 200, 400, 800, 1200, 1600 and 3200 × g. Each of the centrifugation steps lasted 2 min. The supernatant (500 µL) which contained unbound antibodies were then withdrawn. The concentration of antibodies which correlated with the concentration of *C*. *jejuni* was quantified by injecting 75 µL (4 min) of the supernatant fluid over the immobilized primary capture antibody on the SPR sensor surface. Regeneration was carried out using 25 µL of 100 mM HCl, which was injected for the duration of 1 min.

### Detection of *C*. *jejuni* in chicken samples using commercial ELISA kit

A commercial ELISA kit: *Campylobacter* antigen detection (in food) (Diagnostics Automation Inc, CA, USA) was utilised as a comparative method. The kit is based on a double antibody (sandwich) assay utilising specific anti-*Campylobacter* antibodies coated into microwells. The recommendation of the manufacturer was followed in carrying out the experiment. The absorbance was measured at 450 nm (Varioskan Flash Multimode Reader, Thermo Scientific Inc, US).

### Statistics and calculation methods

Values are means ± standard deviation (SD) of triplicate experimental data. All data were analyzed using GraphPad Prism version 5.0. The comparison between two groups was performed using a Student’s t-test, and a comparison between more than two groups was carried out utilizing a one-way analysis of variance with post hoc analysis using the Tukey’s test. P < 0.05 was considered statistically significant.

## Supplementary information

Dataset 1

## Data Availability

All data generated or analyzed during this study are included in this published article (and its Supplementary Information Files).
