# Supplementary material for: Subtractive inhibition assay for the detection of Campylobacter jejuni in chicken samples using surface plasmon resonance
Source: Sci Rep. 2019 Sep 20;9:13642. doi: 10.1038/s41598-019-49672-2 (PMC6754509; doi:10.1038/s41598-019-49672-2)
Supplement: Supplementary file 1 — Dataset 1 [file 41598_2019_49672_MOESM1_ESM.pdf]

Subtractive inhibition assay for the detection of *Campylobacter jejuni* in chicken samples using surface plasmon resonance

Noor Azlina Masdor[1,2]*, Zeynep Altintas[3], Mohd Yunus Shukor[4] and Ibtisam E. Tothill[1]

[1]Cranfield University, Cranfield, Bedfordshire, MK43 0AL England, United Kingdom.
[2]Biotechnology and Nanotechnology Research Center, Malaysian Agricultural Research and Development Institute, MARDI, P. O. Box 12301, 50774 Kuala Lumpur, Malaysia.
[3]Technical University of Berlin, Straße des 17. Juni 124, Berlin 10623, Germany.
[4]Department of Biochemistry, Faculty of Biotechnology and Biomolecular Sciences, Universiti Putra Malaysia, 43400 Serdang, Selangor, Malaysia.

Supporting Information

Fig. 2 data

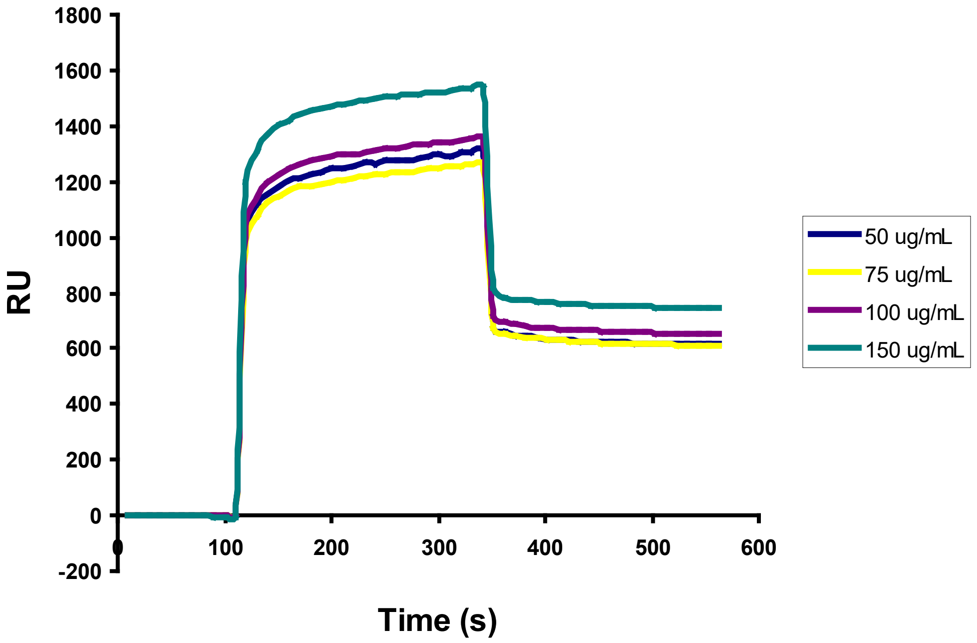

Fig. 2a data

Time	50 ug/mL		75 ug/mL		100 ug/mL		150 ug/mL
9.6	-0.199	9.6	-0.284	9.6	-0.296	9.6	-0.336
19.6	-0.493	19.6	-0.651	19.6	-0.309	19.6	-0.963
29.7	-1.44	29.7	-0.948	29.7	-0.783	29.7	-1.724
39.8	-0.331	39.8	-0.493	39.8	-1.186	39.8	-1.791

49.8	-0.538	49.8	-1.162	49.8	-0.453	49.8	-1.919
59.9	-1.37	59.9	-1.038	59.9	-1.393	59.9	-1.709
70	-0.929	70	-1.416	70	-2.032	70	-2.7
80	-1.331	80	-1.2	80	-2.114	80	-1.591
90.1	-1.677	90.1	-0.961	90.1	-1.962	90.1	-3.166
100.2	-2.382	100.2	-1.673	100.2	-2.674	100.2	-3.266
110.2	-2.367	110.2	-1.886	110.3	-2.164	110.2	-3.299
120.3	1027.598	120.3	983.702	120.3	1043.608	120.3	1195.66
130.4	1118.689	130.4	1075.086	130.4	1147.526	130.4	1314.827
140.4	1155.266	140.4	1122.746	140.5	1200.997	140.4	1371.114
150.5	1184.67	150.5	1148.548	150.5	1229.139	150.5	1405.996
160.6	1202.648	160.6	1166.65	160.6	1245.951	160.6	1423.546
170.6	1213.424	170.6	1181.914	170.7	1262.342	170.6	1439.977
180.7	1227.495	180.7	1187.033	180.7	1274.821	180.7	1454.058
190.8	1233.265	190.8	1193.627	190.8	1280.715	190.8	1463.941
200.8	1246.078	200.9	1201.296	200.9	1288.715	200.9	1473.542
210.9	1247.009	210.9	1206.591	210.9	1296.191	210.9	1479.811
221	1255.185	221	1211.701	221	1297.813	221	1486.169
231.1	1266.347	231.1	1217.658	231.1	1305.47	231.1	1491.835
241.1	1264.835	241.1	1227.896	241.1	1314.875	241.1	1499.356
251.2	1275.345	251.2	1225.476	251.2	1319.203	251.2	1504.825
261.3	1275.118	261.3	1230.379	261.3	1321.759	261.3	1507.531
271.3	1280.091	271.3	1234.955	271.3	1323.718	271.3	1510.164
281.4	1286.009	281.4	1241.294	281.4	1330.492	281.4	1514.127
291.5	1289.036	291.5	1245.592	291.5	1334.625	291.5	1520.631
301.5	1296.94	301.5	1247.766	301.5	1340.118	301.5	1523.221
311.6	1294.204	311.6	1253.869	311.6	1344.319	311.6	1528.119
321.7	1303.847	321.7	1252.437	321.7	1348.594	321.7	1534.28
331.7	1307.088	331.7	1260.089	331.8	1354.222	331.7	1538.014
341.8	1310.652	341.8	1265.165	341.8	1355.416	341.8	1541.242
351.9	672.581	351.9	672.375	351.9	717.396	351.9	818.673
361.9	656.709	361.9	655.346	362	697.028	361.9	783.637
372	647.896	372	645.607	372	688.843	372	775.673
382.1	642.745	382.1	640.671	382.1	682.922	382.1	771.397
392.2	637.769	392.2	635.489	392.2	677.362	392.1	767.883
402.2	634.573	402.2	632.006	402.2	673.96	402.2	764.463
412.3	631.802	412.3	628.981	412.3	670.634	412.3	761.471
422.3	629.726	422.4	626.5	422.4	668.515	422.4	759.516
432.4	627.379	432.4	625.089	432.4	666.606	432.4	758.115
442.5	625.837	442.5	622.997	442.5	663.889	442.5	755.902
452.6	624.793	452.6	620.712	452.6	662.319	452.6	755.561
462.6	622.7	462.6	620.082	462.6	660.473	462.6	754.457
472.7	621.526	472.7	617.894	472.7	659.677	472.7	752.423

482.8	620.183	482.8	617.419	482.8	658.3	482.8	751.496
492.8	619.991	492.8	616.411	492.8	657.16	492.8	749.93
502.9	619.104	502.9	614.686	502.9	656.587	502.9	749.633
513	617.879	513	614.092	513	655.351	513	748.696
523	617.784	523	612.811	0	654.636	523	748.319
533.1	615.895	533.1	612.48	0	653.642	533.1	747.587
543.2	615.295	543.2	611.906	0	652.338	543.2	747.002
553.2	615.073	553.2	611.039	0	652.698	553.2	746.257
563.3	613.942	563.3	610.445	0	650.854	563.3	745.296

Fig. 2b data

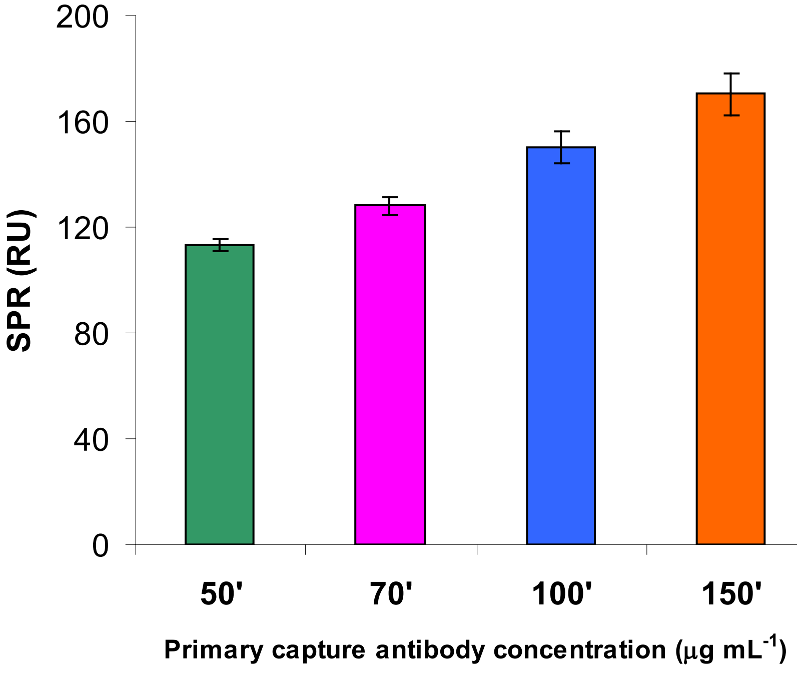

	Avg	stdev
50'	113.445	2.283955
70'	128.025	3.599174
100'	150.215	6.017479
150'	170.275	8.124657

Fig. 2c data

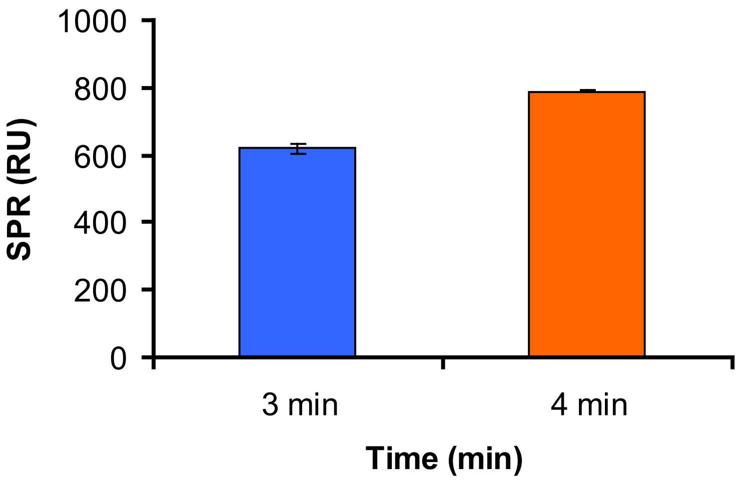

	RU Rep 1	RU Rep 2	RU Rep 3	Avg	stdev
3 min	605.7	635.8	620.75	620.75	15.05
4 min	784.2	793.34	788.77	788.77	4.57

Fig. 3 data

	Average	Stdev
Filter 1	4.22	1.75
Filter 2	35.313	1.87
Centrifugation 1	22.58	1.74
Centrifugation 2	29.715	1.456
Centrifugation 3	37.6	1.625

Fig. 4 data

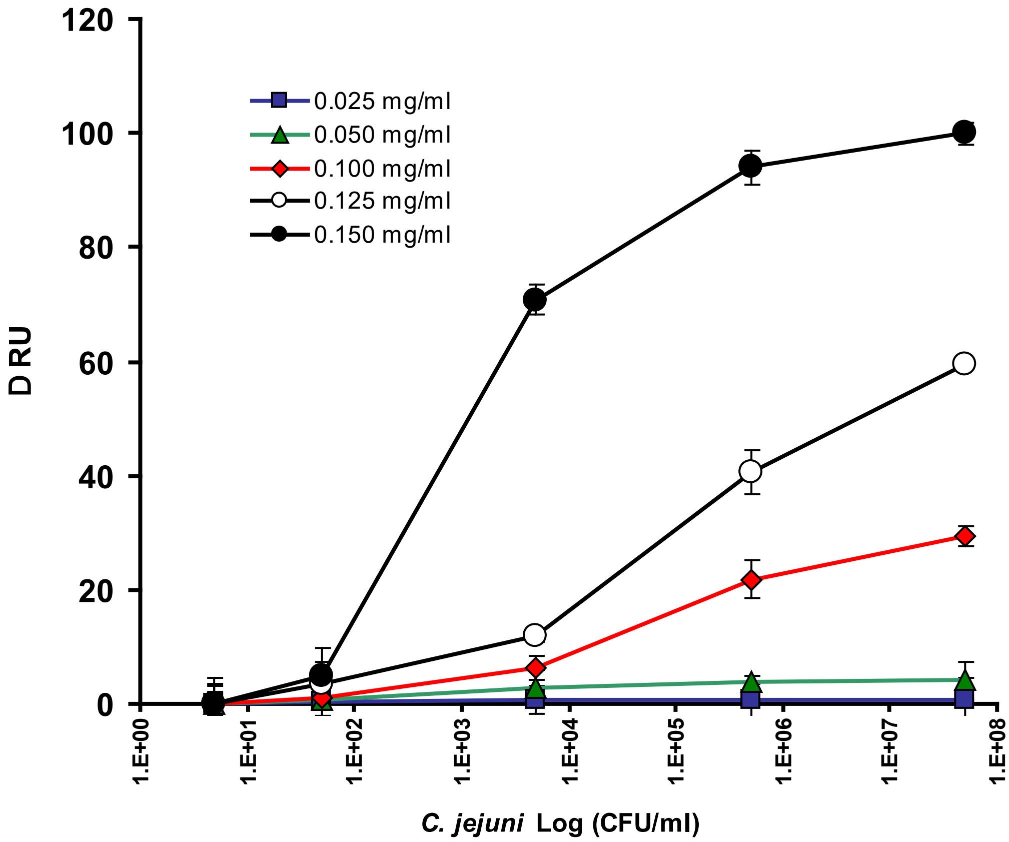

log cfu/ml	0.025 mg/ml	0.050 mg/ml	0.100 mg/ml	0.125 mg/ml	0.15 mg/ml -1.4E-	0.025 mg/ml stdev	0.050 mg/ml stdev	0.1 mg/ml stdev	0.125 mg/ml stdev	0.150 mg/ml stdev
0.698970	0	0	0	0	14	4.481363	3.421269	3.111257	1.965757	1.811257
1.698970	0.33	0.64	1.13	3.5	5.005	3.211333	1.858567	3.395812	4.886108	3.825812
3.698970	0.75	2.71	6.2	11.8	70.745	2.56458	3.116762	2.138668	2.552655	0.838668
5.698970	0.58	3.77	21.8	40.5	93.96	3.003904	1.10218	3.369484	3.054701	3.799484
6.698970	0.83	4.083	29.4	59.5	99.885	3.740788	3.277133	1.750419	1.916259	0.450419

Fig. 5 data

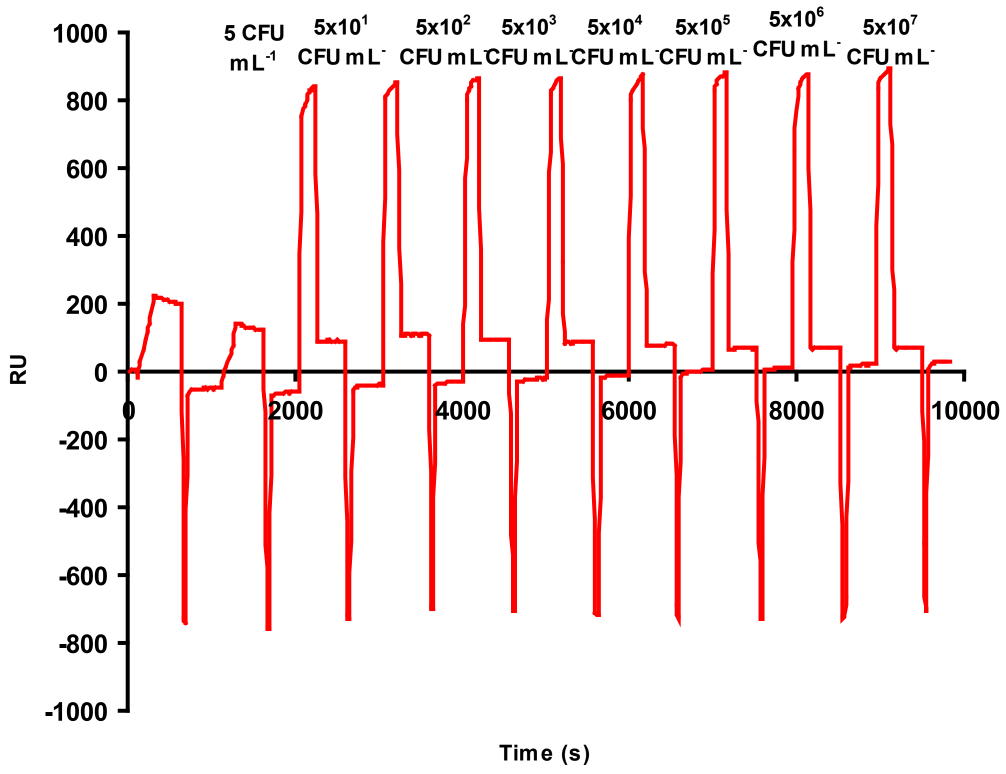

5 CFU mL-1
5x10^1 CFU mL-
5x10^2 CFU mL-
5x10^3 CFU mL-
5x10^4 CFU mL-
5x10^5 CFU mL-
5x10^6 CFU mL-
5x10^7 CFU mL-
RU
Time (s)

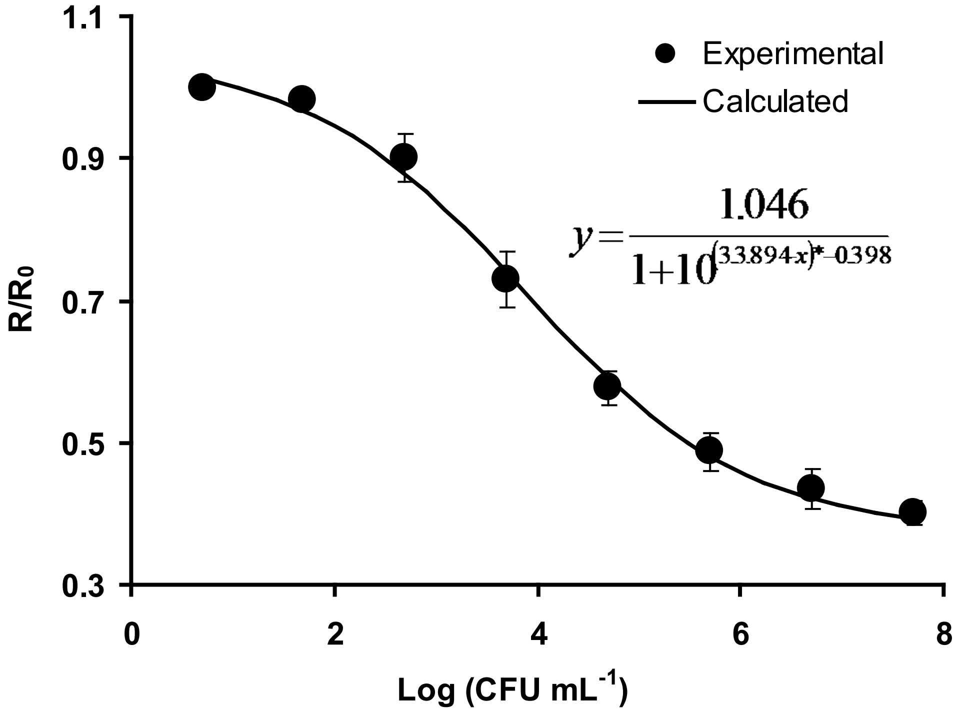

Experimental
Calculated
y = 1.046 / 1+10^((3.3894-x)*-0.398)
R/R0
Log (CFU mL-1)

Experimental Log CFU ml-1	Calculated Log CFU mL-1	Experimental R/Ro	Calculated R/Ro	Std deviation
0.69897	0.69897	0.999998	1.012196	3.76E-06
1.69897	1.067391	0.983167	0.999419	0.004276
2.69897	1.435812	0.901737	0.982345	0.03364
3.69897	1.804233	0.728344	0.959923	0.039105
4.69897	2.172654	0.577084	0.931136	0.023891
5.69897	2.541075	0.487065	0.895233	0.02689
6.69897	2.909496	0.433778	0.852041	0.028327
7.69897	3.277917	0.400593	0.802275	0.015931
	3.646338		0.74771	
	4.01476		0.691043	
	4.383181		0.635414	
	4.751602		0.583742	
	5.120023		0.538152	
	5.488444		0.499719	
	5.856865		0.468544	
	6.225286		0.444036	
	6.593707		0.425242	
	6.962128		0.411099	
	7.330549		0.400609	
	7.69897		0.392911	

Fig. 6 data

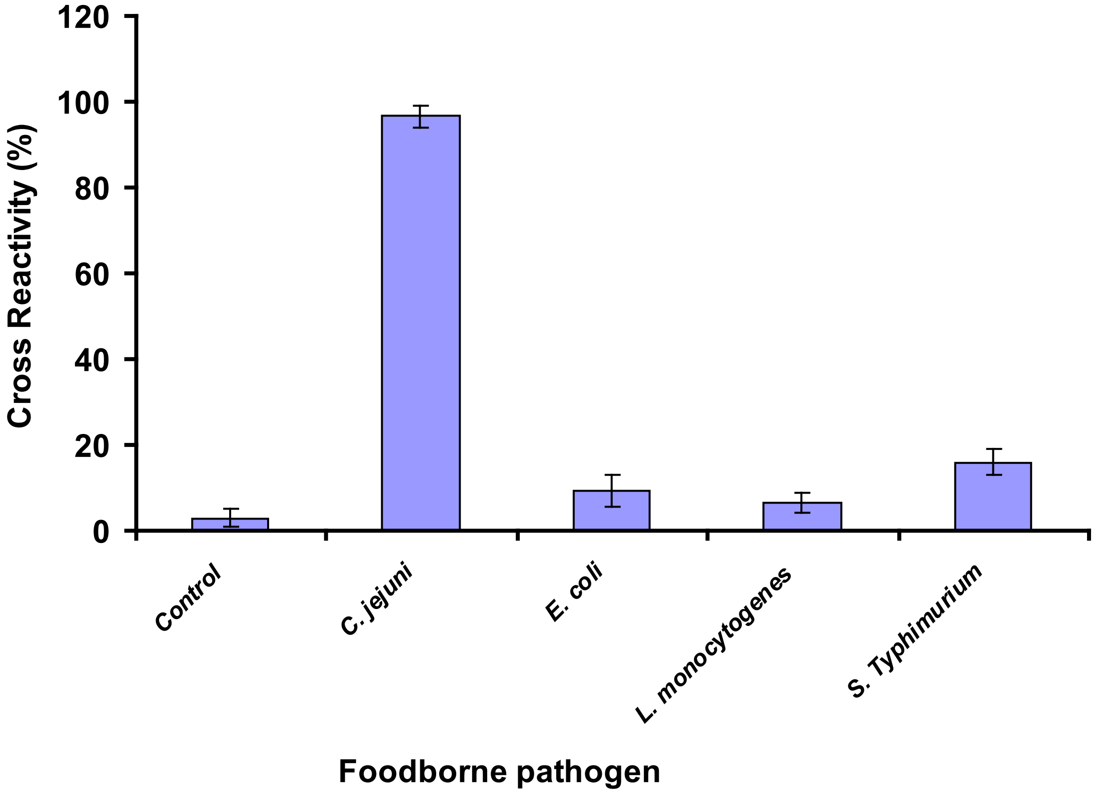

	Average	stdev
Control	2.934222	2.157778
C. jejuni	96.54756	2.616323
E. coli	9.216889	3.862483
L. monocytogenes	6.530222	2.442597
S. Typhimurium	15.97244	2.996209
